# Reflectance Measurements from Aerial and Proximal Sensors Provide Similar Precision in Predicting the Rice Yield Response to Mid-Season N Applications

**DOI:** 10.3390/s23136218

**Published:** 2023-07-07

**Authors:** Telha H. Rehman, Mark E. Lundy, Andre Froes de Borja Reis, Nadeem Akbar, Bruce A. Linquist

**Affiliations:** 1 Department of Plant Sciences, University of California, Davis, CA 95616, USA; melundy@ucdavis.edu (M.E.L.); balinquist@ucdavis.edu (B.A.L.); 2 Division of Agriculture and Natural Resources, University of California, Davis, CA 95618, USA; 3 Division of Plant Science and Technology, University of Missouri, Columbia, MO 65211, USA; areis@missouri.edu; 4 Department of Agronomy, University of Agriculture, Faisalabad 38040, Pakistan; bioworld2020@gmail.com

**Keywords:** rice, nitrogen, sufficiency index, top-dress, UAS, GreenSeeker

## Abstract

Accurately detecting nitrogen (N) deficiency and determining the need for additional N fertilizer is a key challenge to achieving precise N management in many crops, including rice (*Oryza sativa* L.). Many remotely sensed vegetation indices (VIs) have shown promise in this regard; however, it is not well-known if VIs measured from different sensors can be used interchangeably. The objective of this study was to quantitatively test and compare the ability of VIs measured from an aerial and proximal sensor to predict the crop yield response to top-dress N fertilizer in rice. Nitrogen fertilizer response trials were established across two years (six site-years) throughout the Sacramento Valley rice-growing region of California. At panicle initiation (PI), unmanned aircraft system (UAS) Normalized Difference Red-Edge Index (NDRE_UAS_) and GreenSeeker (GS) Normalized Difference Vegetation Index (NDVI_GS_) were measured and expressed as a sufficiency index (SI) (VI of N treatment divided by VI of adjacent N-enriched area). Following reflectance measurements, each plot was split into subplots with and without top-dress N fertilizer. All metrics evaluated in this study indicated that both NDRE_UAS_ and NDVI_GS_ performed similarly with respect to predicting the rice yield response to top-dress N at PI. Utilizing SI measurements prior to top-dress N fertilizer application resulted in a 113% and 69% increase (for NDRE_UAS_ and NDVI_GS_, respectively) in the precision of the rice yield response differentiation compared to the effect of applying top-dress N without SI information considered. When the SI measured via NDRE_UAS_ and NDVI_GS_ at PI was ≤0.97 and 0.96, top-dress N applications resulted in a significant (*p* < 0.05) increase in crop yield of 0.19 and 0.21 Mg ha^−1^, respectively. These results indicate that both aerial NDRE_UAS_ and proximal NDVI_GS_ have the potential to accurately predict the rice yield response to PI top-dress N fertilizer in this system and could serve as the basis for developing a decision support tool for farmers that could potentially inform better N management and improve N use efficiency.

## 1. Introduction

Annually, 60 Tg of nitrogen (N) fertilizer is applied worldwide to produce the three staple food crops (rice, *Oryza sativa*; wheat, *Triticum aestivum*; maize, *Zea mays*), but only 30–50% of applied N is taken up by the crop [1]. This disparity between N fertilizer inputs and outputs negatively impacts the biosphere in many ways. For example, excessive N fertilization can result in nitrate leaching [2], increased greenhouse gas emissions [3], and tailwater eutrophication [4]. Considering the need to intensify global agriculture to feed a growing population [5], these negative impacts will likely worsen unless new practices are developed and adopted that allow farmers to utilize N fertilizer more efficiently.

In California (CA), where rice is predominantly wet-direct-seeded, the recommended N fertilizer practice is to apply the average seasonal crop requirement (typically 150 to 200 kg N ha^−1^) before flooding and planting [6,7]. Linquist et al. [6] reported from 14 on-farm studies that N applied in this manner is used relatively efficiently, leading to an average fertilizer N recovery of 53%. In this system, assessing the crop at panicle initiation (PI) to determine whether the crop requires an additional N fertilizer application as top-dress is recommended [7]. Panicle initiation is considered a critical stage for in-season N management as most of the pre-plant N fertilizer has been taken up by this stage [8], and N fertilizer applied later than PI is less efficiently utilized for grain yield [9]. In CA, top-dress N fertilizer is applied at rates ranging from 22 to 45 kg N ha^−1^, but typically around 34 kg N ha^−1^ [6,7]. While this rate is relatively low, it can still be an important adjustment, particularly when the yield potential may be higher than normal.

Tools currently available to assess crop N status at PI include the Leaf Color Chart (LCC) and the Soil Plant Analysis Development (SPAD) chlorophyll meter [10,11]. Both the LCC and SPAD meter are simple diagnostic tools that have been shown to adequately estimate leaf N content and aid in the development of in-season N recommendations [12,13]. However, the disadvantages of these tools are that they rely on a single leaf sampling method and thus can only assess a small fraction of the field [10]. These constraints have contributed to poor adoption of these tools; thus, top-dress N applications often take place without an assessment of crop N status. This can lead to unnecessary applications of N, potentially causing environmental issues and also reducing grain yields and quality [14], or withholding N applications when they can benefit the crop.

More recently, crop sensors that can measure canopy reflectance-based vegetation indices (VIs) have shown promise in accurately assessing crop N status over larger areas more efficiently [15]. A variety of sensors can be used to record canopy reflectance, including proximal or aerial, active or passive, and utilizing a wide range of spectral resolutions (e.g., multispectral to hyperspectral) [10,16]. Among the numerous VIs currently used in agricultural research, the Normalized Difference Vegetation Index (NDVI) is the most studied. Over the past two decades, numerous reports have demonstrated strong correlations between the NDVI and N status across a wide range of crops, including rice [17,18,19]. Several others have also utilized NDVI to predict rice grain yields [20,21]. However, a known disadvantage of NDVI is that once the crop canopy exceeds a certain threshold, the index saturates [19,22]. Recently, Rehman et al. [23] demonstrated that the degree of NDVI saturation depends on the sensor used, as they found that NDVI measured with a proximal GreenSeeker (GS) (NDVI_GS_) saturates less than an unmanned aircraft system (UAS)-based NDVI (NDVI_UAS_), and is thus more accurate for quantifying crop N status.

To overcome the problem of NDVI saturation, especially from UAS-gathered data, recent studies are increasingly utilizing the Normalized Difference Red-Edge Index (NDRE), which is calculated similarly to the NDVI, except it uses a red-edge band in the place of red. Red-edge radiation can penetrate deeper into the crop canopy than red radiation due to the relatively lower chlorophyll absorption, making the NDRE more sensitive to the chlorophyll content within the entire canopy, and thus less prone to saturation [24,25]. This has been demonstrated by studies that have compared the ability of NDRE and NDVI to assess rice N status using a UAS, with each reporting that the UAS-based NDRE (NDRE_UAS_) saturated less and was thus more sensitive to crop N than NDVI_UAS_ [23,26,27].

While the NDRE_UAS_ and NDVI_GS_ can provide valuable insights into the N status of a developing crop, an absolute VI measurement (using any index) may not accurately reflect the likelihood of the crop to respond to additional N inputs, especially when evaluated across different varieties, environments, and growing seasons [11,28]. To address this issue, an N-enriched area can be established as a reference by applying a non-limiting rate of N [12,15]. Holland and Schepers [29] developed the sufficiency index (SI) by dividing the VI from the field by the VI of an adjacent N-enriched area (can also be referred to as a response index (RI); RI = SI^−1^). The resulting SI generally ranges from 0 to 1, with lower numbers representing stronger N deficiency and increased potential for crop N responsiveness.

Several previous studies have utilized sensor-based SI measurements to inform in-season N management in the major cereal crops (rice, wheat, and maize). In studies that utilized proximal sensors, Clay et al. [30] used NDVI-based SI to quantify wheat N status and estimate the potential for a yield response to additional N fertilizer. Thompson et al. [31] measured NDRE-based SI to develop a predictive model that improved N use efficiency by prescribing in-season N application rates in maize. Cordero et al. [32] and Lu et al. [33] applied a similar method to rice and also reported an improvement in N use efficiency. Raun et al. [34,35] used a NDVI_GS_-based SI (RI) to develop a N fertilization optimization algorithm (NFOA) in wheat to determine crop N needs based on a mass-balance calculation (i.e., optimal N rate required to achieve an estimated yield) [15]. A number of studies applied the NFOA to rice (also using NDVI_GS_) and observed an improvement in agronomic N use efficiency relative to standard farmer practice by producing similar grain yields with less N fertilizer applied [36,37,38,39]. In studies that utilized aerial sensors, Zhang et al. [40] and Thompson et al. [41] measured NDRE-based SI to develop a fertilization algorithm in wheat and maize, respectively, and reported a similar improvement in N use efficiency as the NFOA studies above.

While the results of these studies indicate good utility for sensor-based SI to inform N management in these crops, each of the studies mentioned above based their research on a single sensor (either aerial or proximal), with most of them using NDVI. To the best of our knowledge, no study has compared the ability of the SI to inform N management across aerial and proximal sensors, especially using the NDRE, in any major cereal crop. Considering the lack of studies comparing across sensors, as well as the growing interest in aerial sensors that can assess larger areas more efficiently and with better precision, further research is needed.

The objective of this study was to quantitatively test and compare the ability of aerial NDRE_UAS_ and proximal NDVI_GS_ to predict the rice grain yield response to top-dress N fertilizer applied at PI. The aim was to better understand the potential of these sensors to serve as the basis for developing a decision support tool that could improve farmers top-dress N management and promote better N use efficiency. This objective was pursued via field studies conducted over two years at six different locations using the SI approach to determine N deficiencies.

## 2. Materials and Methods

### 2.1. Site Description

Detailed descriptions of the sites and experimental design used in this study have been published previously [19,23]. Six N response trials (five on-farm and one on-station) were established during the 2017 and 2019 growing seasons (named by proximity to the nearest town or research station and study year), with sites located throughout the Sacramento Valley rice-growing region of CA (Figure S1, Table S1). The on-station site was established at the CA Rice Experiment Station (RES) near Biggs. The Sacramento Valley has a Mediterranean climate characterized by warm and dry conditions during the growing season (May to October). The average air temperature and precipitation during the growing season for the two years of this study were 23.1 °C and 8.0 mm, respectively [42].

### 2.2. Experimental Design and Management

Each trial was arranged as a split-plot randomized complete block design with four replicates. The main plot treatment was pre-plant N fertilizer (6 N rates ranging from 0 to 235 kg N ha^−1^), and the subplot treatment was top-dress N fertilizer applied at PI. Top-dress N fertilizer was broadcast by hand at PI as ammonium sulfate at rates of 0, 25, and 50 kg N ha^−1^ in 2017, and 0 and 34 kg N ha^−1^ in 2019. Phosphorus (P) and potassium (K) were broadcast across all plots at a rate of 45 kg P_2_O_5_ ha^−1^ as triple superphosphate and 50 kg K_2_O ha^−1^ as potassium sulfate to ensure these nutrients were not limiting. The rice was established by wet-direct-seeding, which is the standard practice in CA [43]. In this case, the fields are fertilized following seedbed preparation, flooded, and then soaked seed (variety M-206) is broadcast onto the field by airplane. While seeding rates can vary across fields based on grower management, typical seeding rates range from 170 to 200 kg ha^−1^ [43]. Planting dates were within the expected timeframe for the region (early to mid-May), with the exception of Davis and the RES sites in 2019, where planting was delayed until early June. Herbicide and irrigation management followed common grower practice. No presence of disease or pests was identified in the plots over the course of the experiments.

At physiological maturity, the grain yield was measured by harvesting mature plants from a 1.0 m^2^ quadrat in each subplot. Grains were removed from panicles, cleaned using a seed blower, dried to constant moisture at 60 °C, and then weighed. Final yields are reported at 14% moisture.

### 2.3. Canopy Reflectance Measurements

Prior to top-dress N application, canopy reflectance was measured for each main plot at PI using an aerial and a proximal sensor (Table 1). Before taking canopy reflectance measurements, PI was visually confirmed in the field using the method outlined by Dunn et al. [44]. Canopy closure was achieved by PI in all plots that received N fertilizer; thus, the effect of background water or soil on reflectance measurements was considered negligible in those plots. All canopy reflectance measurements occurred within one hour of solar noon (proximal measurements were collected first, immediately followed by aerial measurements).

#### 2.3.1. Aerial and Proximal Sensors

**Figure 1:**
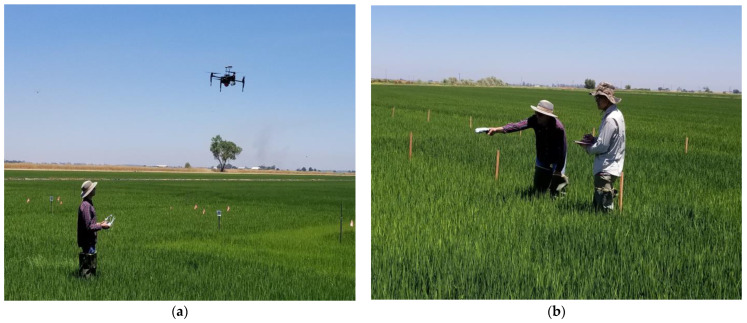
Collecting rice canopy reflectance measurements using (**a**) an aerial MicaSense Red-Edge M sensor mounted to a DJI Matrice 100 and (**b**) a proximal GreenSeeker sensor.

**Figure 2:**
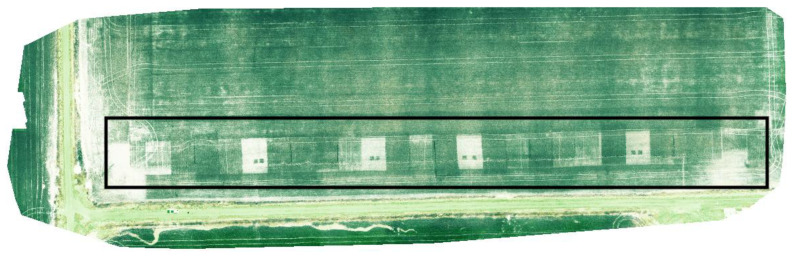
A georeferenced orthomosaic map of a nitrogen response trial depicting the Normalized Difference Red-Edge Index (NDRE).

Two different aerial sensors were used to record canopy reflectance (Table 1, Figure 1a). In both years, the aerial sensor was mounted to a Matrice 100 unmanned aerial vehicle (DJI, Shenzhen, China). Before the beginning of each flight, images of a calibrated reflectance panel were taken to adjust for ambient light conditions. There was also an upwelling light sensor onboard the Matrice 100 that calibrated for incoming irradiance. Okta cloud cover and wind speed readings were taken periodically during every flight using both visual observation and a mobile application and were 0 (completely clear sky with no wind) at all times during the flights. Plot-level canopy reflectance values were converted into unmanned aircraft system (UAS) Normalized Difference Red-Edge Index (NDRE) (NDRE_UAS_) using the formula provided in Table 1. The mobile applications, flight parameters, and computer software used to collect the raw UAS images, process them into georeferenced orthomosaic maps, and extract canopy reflectance values are provided in Table 2. An example of a georeferenced orthomosaic map of a nitrogen response trial depicting NDRE is illustrated in Figure 2.

In both years, the Normalized Difference Vegetation Index (NDVI) was measured using a GreenSeeker (GS) (NDVI_GS_) Handheld Sensor (Trimble Inc., Sunnyvale, CA, USA) (Table 1, Figure 1b). The NDVI_GS_ measurements were taken while steadily walking along the edges of the main plot and holding the GS in the nadir position at a constant height of 1.0 m above the crop canopy and extended 90 cm from the edge of the plot. For each main plot, the final NDVI_GS_ value represented the average of four NDVI_GS_ readings.

#### 2.3.2. Calculating the Sufficiency Index

For every main plot, the sufficiency index (SI) was calculated from the VI values for both indices using the following equation, as outlined by Holland and Schepers [29]:

(1) $$SI=\left({VI}_{N\;treatment}\right)/{(VI}_{N-enriched\;area})$$

At each site, the maximum VI was recorded from the highest pre-plant N rate. The N response curves for yield showed that this N rate was non-limiting and thus served as a valid N-enriched area (results presented below). However, to account for the inherent background variability present within field measurements, the 95th percentile value of the distribution of VI values specific to each site-year was used to serve as the N-enriched area instead of the site-year maximum VI, as recommended by Holland and Schepers [49]. This resulted in SI values greater than 1.00 for 8% of the plot-level observations for each index.

### 2.4. Data Analysis

Data analysis was performed using the statistical program R [50]. The nlme package [51] was used to develop linear mixed-effects models to quantify all of the linear relationships presented in this study. For every mixed-effects model, graphical and numerical summaries were examined to ensure that the resulting model satisfied the assumptions of linear regression. The overall significance of each resulting model’s fixed effects and interactions (if applicable) was tested using a Type III analysis of variance (ANOVA) using the car package [52]. Pseudo R^2^ values were calculated using the MuMIn package [53].

The relationship between grain yield and pre-plant N rate was quantified using a quadratic linear mixed-effects model, where:

(2) $$\begin{array}{l} Grain\;Yield=\\ fixed=preplant\;N\;rate+{\left(preplant\;N\;rate\right)}^{2},\\ random=\sim preplant\;N\;rate+{\left(preplant\;N\;rate\right)}^{2}|site-year \end{array}$$

The resulting model fixed-effects coefficients were used to describe the relationship for all sites, collectively, and the model random effects coefficients were used to describe the relationship for each site individually. The vertex was quantified to determine the optimal pre-plant N rate (i.e., N rate required for maximum yields) for each site individually and across all sites on average.

At each site, the mean yield response to top-dress N fertilizer was calculated for every subplot by subtracting the yield without a top-dress N application from the yield of the subplot that received top-dress N. For the 2017 sites, a significant difference was not observed between the 25 and 50 kg N ha^−1^ top-dress N rates; thus, an average yield response of the two subplots was calculated. The yield response from the subplots was then averaged across the four replications of each pre-plant N treatment at each site-year and the standard deviation was calculated.

The relationship between each SI and pre-plant N rate was quantified using quadratic linear mixed-effects models, where:

(3) $$\begin{array}{l} Sufficiency\;Index(SI)=\\ fixed=preplant\;N\;rate+{\left(preplant\;N\;rate\right)}^{2},\\ random=\sim preplant\;N\;rate+{\left(preplant\;N\;rate\right)}^{2}|site-year \end{array}$$

The vertex of the resulting models was quantified to determine the pre-plant N rate at the point of saturation for each index.

The relationship between each SI, top-dress N rate, and grain yield was quantified using linear mixed-effects models, where:

(4) $$\begin{array}{l} Grain\;Yield=\\ fixed=SI+top-dress\;N\;rate+SI*top-dress\;N\;rate,\\ random=\sim top-dress\;N\;rate|site-year \end{array}$$

Both the SI and the top-dress N rate were included in the models as continuous variables. From these models, the grain yield response to top-dress N fertilizer was defined as the increase in the estimated final grain yield as a function of top-dress N compared to the control (0 N top-dress). Within the range of top-dress N rates included in this study (25–50 kg N ha^−1^), a significant difference in the grain yield response was not detected. Considering this lack of a significant difference among the rates, the grain yield response to top-dress N was estimated at a single rate of 34 kg N ha^−1^, which is the midpoint of study rates and a typical top-dress N rate in CA [7].

Using the coefficients from the linear mixed-effects models presented in Equation (4), estimated marginal means were calculated for grain yield across the range of SI values observed in the study. The effect of adding 34 kg N ha^−1^ at PI was compared to not adding N fertilizer at PI across the range of SI values using the contrast() function [54]. For each SI value, the probability of realizing a positive yield response (i.e., response > 0 Mg ha^−1^) by applying 34 kg N ha^−1^ at PI was determined via a one-sided pairwise comparison of the effect size and an associated t-test. The average yield response to top-dress N fertilizer without considering the SI was also derived by applying the contrast() function to the estimated marginal means.

## 3. Results

### 3.1. Crop Response to N Fertilizer

**Figure 3:**
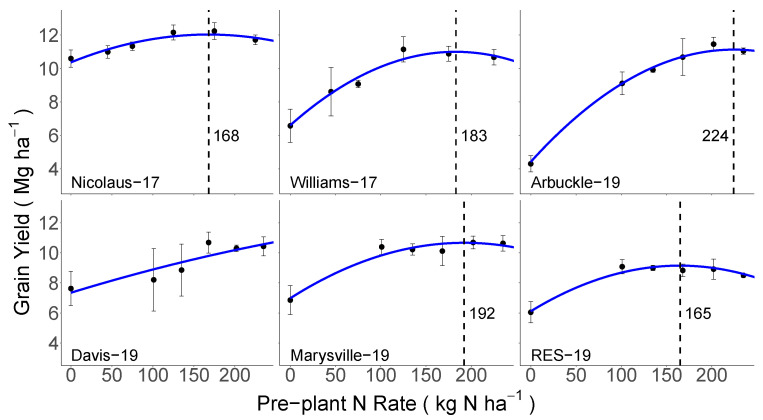
The relationship between the pre-plant N rate and rice grain yield without top-dress N for each site-year, as described by a quadratic linear mixed-effects model. The vertical dashed lines and values reported represent the optimal pre-plant N rate for each site (i.e., the N rate required to achieve maximum yields). Note: the relationship did not level-off at the Davis-19 site within the range of pre-plant N rates used in this study, and thus no pre-plant N rate is marked.

Across the six site-years, where top-dress N was not applied, minimum grain yields ranged widely, from 4.3 to 10.6 Mg ha^−1^, and were always observed in the 0 N pre-plant treatment (Figure 3). Maximum grain yields ranged from 9.1 to 12.2 Mg ha^−1^. Based on the mixed-effects model presented in Equation (2), yields increased with the increasing pre-plant N rate at each site to a maximum, and then leveled off or decreased at higher N rates. The only exception was at the Davis-19 site, where the modeled relationship did not level off within the range of pre-plant N rates. However, numerically, maximum yields at this site were achieved at the 168 kg N ha^−1^ rate. Based on the model results, the optimal N rate (i.e., N rate required to achieve maximum yields) ranged from 165 to 224 kg N ha^−1^ and averaged 200 kg N ha^−1^ (Figure S2) across all sites.

**Figure 4:**
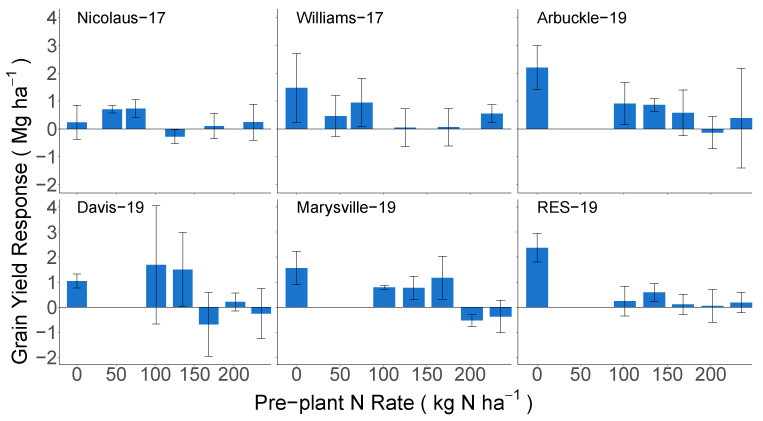
The average grain yield response to top-dress N fertilizer applied at the panicle initiation (PI) rice growth stage. The error bars represent the standard deviation.

Applying top-dress N at PI led to an increase in grain yields in the lower pre-plant N rates at all sites, but as the pre-plant N rate increased, the yield response to top-dress N decreased (Figure 4). For example, when top-dress N was added in the 0 N pre-plant treatment, yields increased from 0.1 to 2.4 Mg ha^−1^ and averaged 0.9 Mg ha^−1^. In contrast, at the highest pre-plant N rate, top-dress N applications led to both yield increases (0.7 Mg ha^−1^ at Williams-17) and decreases (−0.4 Mg ha^−1^ at Marysville-19), with an average yield response of 0.1 Mg ha^−1^.

### 3.2. Canopy Reflectance and Linear Relationships between Pre-Plant N Rate and SI

**Figure 5:**
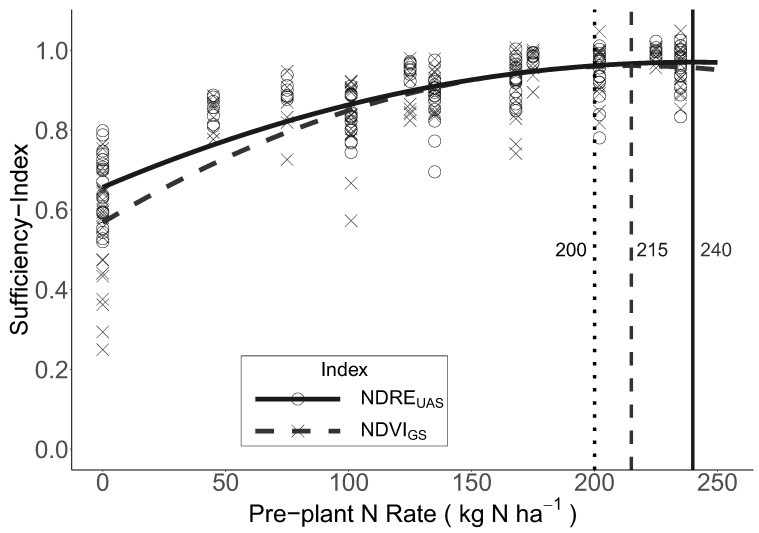
The relationships between the pre-plant N rate and unmanned aircraft system (UAS) Normalized Difference Red-Edge Index (NDRE_UAS_) sufficiency index (SI) and GreenSeeker (GS) Normalized Difference Vegetation Index (NDVI_GS_) SI measured at panicle initiation (PI), as described by quadratic linear mixed-effects models. The solid and dashed vertical lines at 240 and 215 kg N ha^−1^, respectively, represent the N rate where the relationships saturated (i.e., the vertices of the quadratic models). The dotted vertical line at 200 kg N ha^−1^ represents the optimal pre-plant N rate for all sites (i.e., the N rate required to achieve maximum yields on average), as derived from Figure S2.

At every site, for both the NDRE_UAS_ and the NDVI_GS_, the VI and SI increased with the increasing pre-plant N rate to a point, and then tended to plateau at the higher N rates (Figure 5, see Tables S2–S4 for site details). Overall, SI ranged from 0.52 to 1.03 and 0.25 to 1.05 for the NDRE_UAS_ and the NDVI_GS_, respectively.

Quadratic mixed-effects models were developed to describe the relationships between the pre-plant N rate and SI for both the NDRE_UAS_ and NDVI_GS_ (Equation (3), Figure 5). The SI for NDRE_UAS_ saturated (i.e., reached the vertex) at 240 kg N ha^−1^, while the NDVI_GS_ saturated at 215 kg N ha^−1^. Based on the models, these points of saturation corresponded to a SI of 0.97 and 0.96 for the NDRE_UAS_ and the NDVI_GS_, respectively. As mentioned earlier, the optimal pre-plant N rate averaged 200 kg N ha^−1^ across all sites (Figure S2). Importantly, both the NDRE_UAS_ and NDVI_GS_ saturated beyond 200 kg N ha^−1^, indicating that both indices are sensitive to differences in plant N within system-relevant N rates for in-season management.

### 3.3. Relationship between SI and Grain Yield Response to Top-Dress

Mixed-effects models were developed to describe the relationship between the top-dress N rate, the SI, and grain yield (Equation (4), Table 3). The R^2^, slope, and mean model standard error of the resulting mixed-effect models were similar for the two SIs. Each model explained 74% of the variability in grain yield. In both cases, the majority of this variability was explained by the model fixed-effects (SI, top-dress N rate, and their interaction). For the NDRE_UAS_, the fixed-effects explained 50%, while the site-year random effects explained 24%. In the case of NDVI_GS_, the proportion of variability explained by the fixed and random effects was 44% and 30%, respectively.

### 3.4. Estimating Crop Yield Response to Top-Dress N Fertilizer via SI

**Figure 6:**
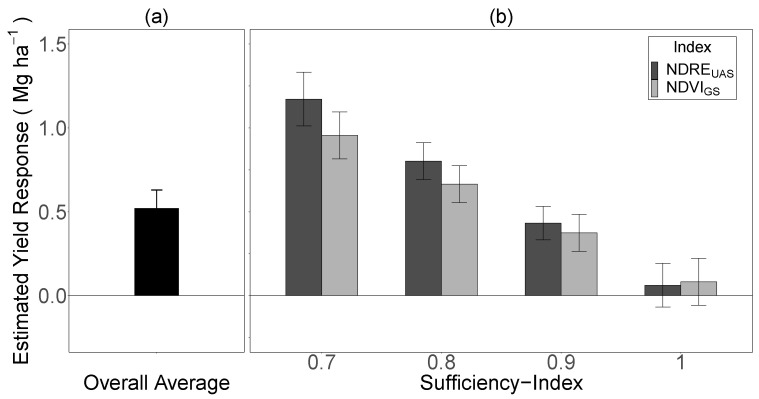
The estimated rice grain yield response to top-dress N fertilizer applied at the panicle initiation (PI) growth stage using a rate of 34 kg N ha^−1^ (typical grower rate): (**a**) overall average (averaged across SI 0.70 to 1.00), and (**b**) for the unmanned aircraft system (UAS) Normalized Difference Red-Edge Index (NDRE_UAS_) sufficiency index (SI) and the GreenSeeker (GS) Normalized Difference Vegetation Index (NDVI_GS_) SI at specific SI values corresponding to the typical management range (i.e., pre-plant N rates of 150 to 200 kg N ha^−1^), as estimated by linear mixed-effects models. The error bars represent the standard error around the estimated grain yield response.

To quantify the benefit of using a SI to estimate the yield response to top-dress N fertilizer within typical management scenarios, the results from the above models were confined to a SI range of 0.70 to 1.00, which represents the range of SI values farmers would most likely measure from their fields under normal pre-plant fertilization rates (150 to 200 kg N ha^−1^). The SI values between 0.70 and 1.00 represented 96% and 92% of the observations for pre-plant rates between 150 to 200 kg N ha^−1^ for the NDRE_UAS_ and the NDVI_GS_, respectively (Figure 5). Across sites, the average yield response to a 34 kg N ha^−1^ top-dress applied at PI within the range of normal pre-plant N rates and without any information regarding crop N status (i.e., SI) was 0.52 ± 0.11 Mg ha^−1^ (Figure 6a). In comparison, yield responses to top-dress N applications were more precisely differentiated when the SI at PI was considered. For example, the estimated yield response ranged from 1.17 to 0.06 Mg ha^−1^ for the NDRE_UAS_ and from 0.96 to 0.08 Mg ha^−1^ for the NDVI_GS_ (Figure 6b), representing a 113% and 69% improvement in yield response differentiation, respectively, compared to the average yield response of 0.52 Mg ha^−1^. Furthermore, the data illustrated that applying top-dress N fertilizer at PI may not be economically viable and could result in reduced yields when SI = 1.0, measured via both the NDRE_UAS_ and the NDVI_GS_.

The probability of realizing a positive yield response (i.e., response > 0 Mg ha^−1^) depended on the SI value measured prior to N fertilizer application at PI (Table 4). For both the NDRE_UAS_ and the NDVI_GS_, as the SI decreased, the probability of a positive yield response increased. A 95% probability of realizing a significant yield response occurred at a similar level for both SIs (0.97, NDRE_UAS_; 0.96, NDVI_GS_), and the estimated grain yield responses at this probability level were 0.19 and 0.21 for the NDRE_UAS_ and the NDVI_GS_, respectively.

## 4. Discussion

### 4.1. Grain Yields and Response to Pre-Plant N Fertilizer

The maximum yields observed in this study (Figure 3) were within 85% of the yield potential for this region [55], which should be achievable with good management practices and suggests that site yields were not limited by biotic or abiotic stresses [56]. Overall, the maximum grain yields were greater than 10.7 Mg ha^−1^, with the only exception being RES-19, where the yields were 9.1 Mg ha^−1^. In CA, most rice is planted in May [43], and the late planting date (12 June) at RES-19 may have contributed to a reduced yield potential and the lower yields observed there. Importantly, at all sites, maximum attainable yields in response to pre-plant N were achieved, as indicated by the decreasing or leveling off of yields at the higher N rates (Figure 3; Figure S2). This indicates that at each site, there were plots that were not N-limited, and thus were valid N-enriched areas for developing a SI.

While maximum yields were generally similar across sites, the optimal pre-plant N rate varied considerably, ranging from 165 to 224 kg N ha^−1^ (Figure 3) and averaging 200 kg N ha^−1^ (Figure S2). Importantly, this variability in the optimal N rate illustrates that N requirements can differ across sites due to the variability in soils, management, and microclimates, and highlights the need for tools that can accurately assess crop N status and help farmers make informed top-dress N management decisions.

### 4.2. Informing Top-Dress N Management with Aerial and Proximal Sensors

A key question regarding these sensors is whether they have the necessary sensitivity to detect N deficiencies so that the SI developed from them does not saturate at levels of crop N status that are deficient. Both SIs saturated (240 and 215 kg N ha^−1^ for the NDRE_UAS_ and NDVI_GS_, respectively; Figure 5) beyond the N rate required to achieve maximum yields (200 kg N ha^−1^), indicating that these SIs have the necessary sensitivity required to detect N deficiencies, and aligns with the findings of a previous study [23].

Relative to applying top-dress N without a determination of crop N status at PI (Figure 6a), using the sensors to determine the SI improved the yield response differentiation to top-dress N fertilizer by 113% and 69% for the NDRE_UAS_ and the NDVI_GS_, respectively (Figure 6b). This suggests that measuring the SI via NDRE_UAS_ or NDVI_GS_ could potentially improve the N use efficiency in this system by identifying fields that are N-deficient and require N fertilizer to optimize yields, or are N-sufficient, indicating that a N application is not required and may reduce yields and/or result in the aforementioned negative environmental consequences [14,57].

In California and other global rice production systems, the recommended approach to managing N fertilizer is to apply the total recommended N rate early in the season and assess the need to apply a top-dress mid-season [7,58,59,60,61]. The data provided here indicated that developing a SI based on either the NDRE_UAS_ or NDVI_GS_ is a promising way to make this assessment. It is important to note that using this approach, the decision to apply a top-dress of N will ultimately depend on the SI of the crop and the cost of applying top-dress N fertilizer. For example, based on the average cost of applying top-dress N fertilizer and the market value of medium-grain rice [62], a yield response ≥ 0.30 Mg ha^−1^ would be required for top-dress N applications to be economically viable. This suggests that, while biologically significant yield responses (i.e., response > 0 Mg ha^−1^; *p* < 0.05) were observed at SI values ≤ 0.97 and 0.96 for the NDRE_UAS_ and the NDVI_GS_, respectively (Table 4), lower SI values would be required to indicate crop N deficiency to a level that the yield response to top-dress N fertilizer would be large enough to justify the costs associated with application. To illustrate this, based on the data presented here, a SI ≤ 0.89 and 0.86 (measured via NDRE_UAS_ and NDVI_GS_, respectively) would be necessary to ensure that the magnitude of response is sufficient to overcome the cost of application.

### 4.3. Comparison between Aerial NDRE_UAS_ and Proximal NDVI_GS_

Interestingly, all metrics evaluated in this study indicated that the two sensors used to measure the NDRE_UAS_ and the NDVI_GS_ performed similarly with respect to detecting N deficiency and predicting the rice yield response to top-dress N fertilizer at PI (Figure 5 and Figure 6b; Table 3 and Table 4). This suggests that farmers may have the flexibility to choose either an aerial or a proximal sensor when making top-dress N decisions in this system as they could expect a similar crop yield response at a given SI value measured via either the NDRE_UAS_ or the NDVI_GS_. The similarity across the two sensors seen here supports a previous study showing that the NDRE_UAS_ and NDVI_GS_ measured at PI have similar sensitivities for quantifying rice N status and predicting grain yield [23]. While providing a theoretical explanation for the similarity across the two sensors is beyond the scope of this study, a possible explanation could be that despite being a red-based index, the closer proximity and active light source of the sensor used to measure the NDVI_GS_ increased the sensitivity to crop N to such a degree that it exhibited a similar level of performance as the red-edge-based NDRE_UAS_ [63]. In partial support of this hypothesis, a number of studies have shown that proximally measured NDVI is more sensitive to crop N status than NDVI measured aerially [23,64,65].

To our knowledge, no other study has compared the ability of aerial and proximal sensors to predict the yield response to in-season top-dress N fertilizer across any of the major cereal crops (rice, wheat, and maize). One study measured NDVI-based SI from aerial and proximal sensors to develop response probabilities to in-season N fertilization in barley (*Hordeum vulgare*), but the researchers did not observe a difference between the sensors and thus reported their results as an average of the two methods [48]. Previous studies that have compared across sensors have mostly focused on the ability of these sensors to quantify in-season crop N status. For example, Rehman et al. [23] compared the sensitivity of aerial NDRE_UAS_ and proximal NDVI_GS_ to quantity N status in rice and found that both performed similarly. Sumner et al. [65] reported a similar result in maize as they found that proximal NDVI (measured with a Yara N-Sensor) and aerial NDRE_UAS_ were both more sensitive to changes in N fertilizer rate than aerial NDVI_UAS_. In a study that only measured NDVI, Zheng et al. [64] reported that proximal NDVI (measured with a hyperspectral sensor) provided a better assessment of rice N status than NDVI_UAS_ due to relatively less saturation. Despite the limited number of comparable studies, when taken together, the results of the current study and previous research indicate that measuring the SI using these sensors, particularly those SIs that have the appropriate sensitivity to crop N, is a useful approach to detect N deficiency and inform in-season N management. Furthermore, the results of the current study suggest that the NDRE_UAS_ and the NDVI_GS_ can be used interchangeably in this regard.

## 5. Conclusions

There are several well-developed methods for assessing in-season crop N needs using sensors. The question is whether different sensors can provide equally precise measurements for these methods. This study addressed that question by quantitively testing and comparing the ability of aerial and proximal sensors to assess the N status of rice and predict the yield response to top-dress N fertilizer applied at PI. The results indicated that as the crop SI measured via the NDRE_UAS_ and the NDVI_GS_ at PI decreased, the yield response to top-dress N fertilizer and the probability of realizing a positive yield response both increased. Interestingly, the results showed that despite the differences between the sensors used for measurement, both the NDRE_UAS_ and the NDVI_GS_ can be used to assess the in-season crop N status and predict the grain yield response to top-dress N with similar precision. The similarity between the NDRE_UAS_ and the NDVI_GS_ with respect to informing top-dress N management is promising and could potentially be an advantage for end-users as it would afford them the flexibility to select the sensor that is most suited to their needs. While the information provided here serves as the basis for developing and refining a tool to make mid-season N management decisions in this system, additional on-farm studies are required to test and validate the results and thresholds of N deficiency presented here, especially on a larger spatial scale.

## Figures and Tables

**Table 1:** Summary of the aerial and proximal sensors used to measure the Normalized Difference Red-Edge Index (NDRE) and the Normalized Difference Vegetation Index (NDVI) at the panicle initiation (PI) rice growth stage.

Vegetation Index	Sensor Type	Year	Sensor	Light Source	Spectral Band	Central Wavelength (nm)	Bandwidth ^†^ (nm)	Formula	Reference
**NDRE**	Aerial	2017	SlantRange 3P	Passive	Red-Edge	710	20	$\frac{\left(Near\;IR-Red\;Edge\right)}{\left(Near\;IR+Red\;Edge\right)}$	[45]
					Near Infrared	850	100		
		2019	MicaSense Red Edge-M	Passive	Red-Edge	717	10		
					Near Infrared	840	40		
**NDVI**	Proximal	Both	GreenSeeker	Active	Red	670	10	$\frac{\left(Near\;IR-Red\right)}{\left(Near\;IR+Red\right)}$	[46]
					Near Infrared	780	10		

**^†^** Full width at half maximum.

**Table 2:** Summary of mobile applications and flight parameters used to collect unmanned aircraft system (UAS) raw imagery in the field, and the computer software used to process the raw imagery into orthomosaic maps and extract canopy reflectance values.

	Mobile Application	Flight Parameters		Computer Software			
Year	Flight Mission Planning	Image Overlap ^†^ (%)	Flight Altitude ^§^ (meters)	Image Processing	Orthomosaic Spatial Resolution ^‡^ (cm pixel^−1^)	Reflectance Value Extraction	Reference
**2017**	Drone Deploy	55	117	SlantView	4.8	SlantView	
**2019**	Pix4D Capture	85	50	Pix4D	3.5	QGIS	[47,48]

^†^ Side and front/back. ^§^ Above ground level. ^‡^ Average ground sampling distance.

**Table 3:** Model parameters of the linear mixed-effects models developed to describe the relationship between the unmanned aircraft system (UAS) Normalized Difference Red-Edge Index (NDRE_UAS_) sufficiency index (SI) and the GreenSeeker (GS) Normalized Difference Vegetation Index (NDVI_GS_) SI, top-dress N fertilizer applied at the panicle initiation (PI) growth stage, and grain yield.

	NDRE_UAS_	NDVI_GS_
Number of Site-Years	6	6
Number of Observations	336	336
Range of SI	0.52–1.03	0.25–1.05
R^2^		
Fixed-Effects	0.50	0.44
Random Effects	0.24	0.30
Entire Model	0.74	0.74
Slope (Mg ha^−1^) ^†^	−0.37 per 0.1 SI	−0.29 per 0.1 SI
Mean Model Standard Error (Mg ha^−1^) §	±0.15	±0.19

^†^ Decrease in the estimated grain yield response to top-dress N per 0.1 unit increase in SI. § Averaged across the range of SI observations.

**Table 4:** The probability of observing a positive yield response to top-dress N fertilizer applied at the panicle initiation (PI) rice growth stage, the model-estimated yield response at each probability level, and the corresponding sufficiency index (SI) (unmanned aircraft system (UAS) Normalized Difference Red-Edge Index (NDRE_UAS_) and GreenSeeker (GS) Normalized Difference Vegetation Index (NDVI_GS_)), as derived from linear mixed-effects models. The values in parentheses represent the standard error of the estimated response.

Positive Yield Response (Response > 0.0 Mg ha^−1^)					
NDRE_UAS_			NDVI_GS_		
Probability (%)	Model-Estimated Response (Mg ha^−1^)	Corresponding SI	Probability (%)	Model-Estimated Response (Mg ha^−1^)	Corresponding SI
**69**	0.06 (±0.13)	1.00	**73**	0.08 (±0.14)	1.00
**80**	0.11 (±0.12)	0.99	**80**	0.12 (±0.13)	0.99
**85**	0.12 (±0.12)	0.98	**85**	0.14 (±0.13)	0.98
**90**	0.15 (±0.12)	0.98	**90**	0.16 (±0.13)	0.97
**95**	0.19 (±0.11)	0.97	**95**	0.21 (±0.12)	0.96

## Data Availability

The data and R script used to perform the analysis and generate this manuscript are openly available on GitHub and archived in Zenodo at https://doi.org/10.5281/zenodo.7964241.

## Supplementary Materials

The following supporting information can be downloaded at: https://www.mdpi.com/article/10.3390/s23136218/s1, Figure S1: A map of N response trial sites established during the 2017 and 2019 growing seasons in the Sacramento Valley rice-growing area of California, USA. Figure S2: The relationship between pre-plant N rate and rice grain yield without top-dress N, as described by a quadratic linear mixed-effects model. The vertical dashed line at 200 kg N ha^−1^ represents the optimal pre-plant N rate for all sites (i.e., the N rate required to achieve maximum yields on average). Table S1: Soil descriptions and selected properties of each N response trial site-year located throughout the Sacramento Valley, California. Table S2: Descriptive statistics (minimum, maximum, and mean) of the GreenSeeker (GS) Normalized Difference Vegetation Index (NDVI_GS_) and the unmanned aircraft system (UAS) Normalized Difference Red-Edge Index (NDRE_UAS_) vegetation index (VI) and sufficiency index (SI) measured at the panicle initiation (PI) rice growth stage from N response trial sites established during the 2017 growing season. Table S3: Descriptive statistics (minimum, maximum, and mean) of the GreenSeeker (GS) Normalized Difference Vegetative Index (NDVI_GS_) vegetation index (VI) and sufficiency index (SI) measured at the panicle initiation (PI) rice growth stage from N response trial sites established during the 2019 growing season. Table S4: Descriptive statistics (minimum, maximum, and mean) of the unmanned aircraft system (UAS) Normalized Difference Red-Edge Index (NDRE_UAS_) vegetation index (VI) and sufficiency index (SI) measured at the panicle initiation (PI) rice growth stage from N response trial sites established during the 2019 growing season.
