# Supplementary material for: Reflectance Measurements from Aerial and Proximal Sensors Provide Similar Precision in Predicting the Rice Yield Response to Mid-Season N Applications
Source: Sensors (Basel). 2023 Jul 7;23(13):6218. doi: 10.3390/s23136218 (PMC10346761; doi:10.3390/s23136218)
Supplement: Supplementary file 1 [file sensors-23-06218-s001.zip › sensors-2316296-supplementary.pdf]

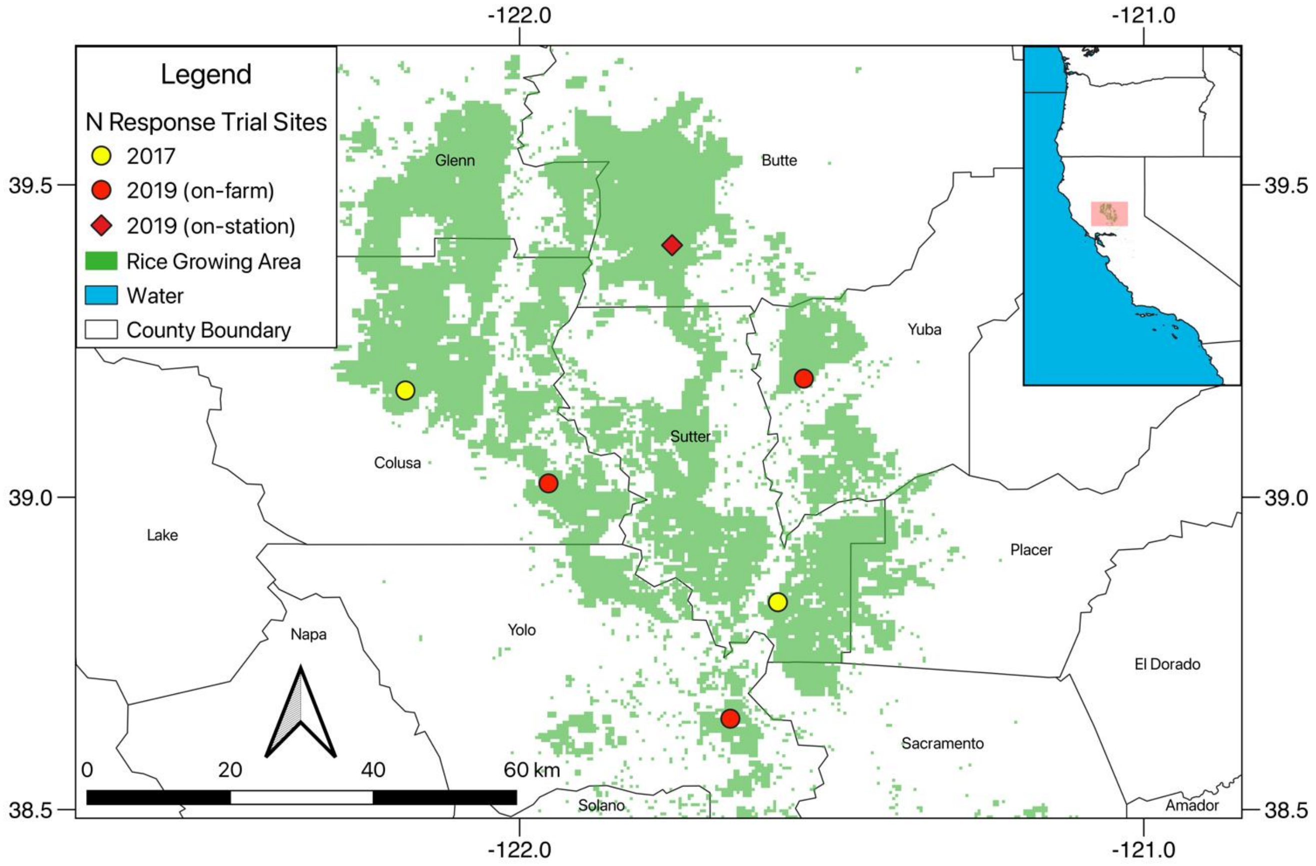

Figure S1. A map of N response trial sites established during the 2017 and 2019 growing seasons in the Sacramento Valley rice growing area of California, USA.

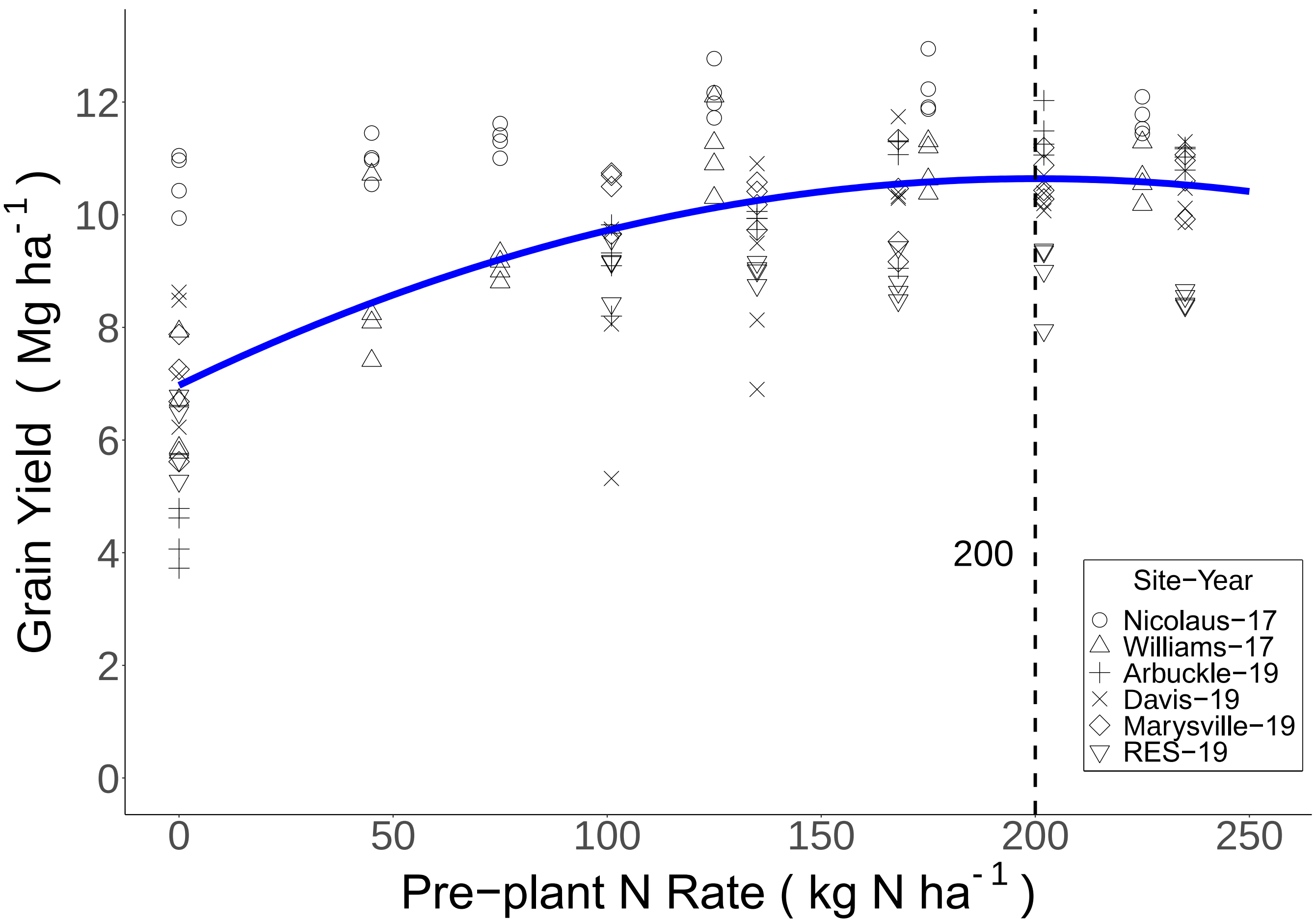

Figure S2. The relationship between pre-plant N rate and rice grain yield without top-dress N as described by a quadratic linear mixed-effects model. The vertical dashed line at 200 kg N ha^{-1} represents the optimal pre-plant N rate all sites (i.e. the N rate required to achieve maximum yields on average).

Table S1. Soil descriptions and selected properties of each N response trial site-year located throughout the Sacramento Valley, California.

Site-Year	Soil Series	Taxonomic Classification	Texture (%)			Organic Carbon (g kg^{-1})	Total Nitrogen (g kg^{-1})	pH
			Sand	Silt	Clay			
Nicolaus-17	Capay	Fine, smectitic, thermic Typic Haploxererts	19	36	45	15.1	1.2	5.5
Williams-17	Willows	Fine, smectitic, thermic Sodic Endoaquerts	21	39	40	17.5	1.5	5.0
Arbuckle-19	Clear Lake	Fine, smectitic, thermic Xeric Endoaquerts	8	38	55	19.9	1.6	6.3
Davis-19	Sycamore	Fine-silty, mixed, super active, nonacid, thermic Mollic Endoaquepts	9	38	53	19.8	1.8	6.3
Marysville-19	San Joaquin	Fine, mixed, active, thermic Abruptic Durixeralfs	35	41	24	15.4	1.2	4.7
RES-19	Esquon-Neerdobe	Fine, smectitic, thermic Xeric Epiaquerts	30	26	44	13.8	1.1	5.3

Table S2. Descriptive statistics (minimum, maximum, and mean) of GreenSeeker (GS) Normalized Difference Vegetation Index ($NDVI_{GS}$) and unmanned aerial system (UAS) Normalized Difference Red-Edge Index ($NDRE_{UAS}$) vegetation index and Sufficiency-Index measured at the panicle initiation rice growth stage from N response trial sites established during the 2017 growing season.

Vegetation Index

Pre-plant N rate (kg N ha^{-1})	$NDVI_{GS}$				$NDRE_{UAS}$			
	Nicolaus-17		Williams-17		Nicolaus-17		Williams-17	
	min – max	mean	min – max	mean	min – max	mean	min – max	mean
**0**	0.49 – 0.52	0.51	0.36 – 0.45	0.41	0.54 – 0.61	0.58	0.49 – 0.58	0.54
**45**	0.61 – 0.68	0.64	0.69 – 0.72	0.70	0.65 – 0.67	0.66	0.63 – 0.69	0.65
**75**	0.57 – 0.72	0.66	0.67 – 0.77	0.70	0.67 – 0.72	0.69	0.69 – 0.71	0.70
**125**	0.66 – 0.68	0.67	0.67 – 0.79	0.74	0.73 – 0.74	0.73	0.72 – 0.76	0.74
**175**	0.71 – 0.79	0.75	0.77 – 0.81	0.80	0.75 – 0.76	0.76	0.76 – 0.78	0.77
**225**	0.78 – 0.79	0.79	0.77 – 0.82	0.79	0.75 – 0.78	0.76	0.77 – 0.79	0.78
**All**	0.49 – 0.79	0.67	0.36 – 0.82	0.69	0.54 – 0.78	0.70	0.49 – 0.79	0.70

Sufficiency-Index

Pre-plant N rate (kg N ha^{-1})	$NDVI_{GS}$				$NDRE_{UAS}$			
	Nicolaus-17		Williams-17		Nicolaus-17		Williams-17	
	min – max	mean	min – max	mean	min – max	mean	min – max	mean
**0**	0.62 – 0.65	0.64	0.44 – 0.56	0.50	0.71 – 0.80	0.76	0.63 – 0.74	0.69
**45**	0.78 – 0.87	0.82	0.86 – 0.89	0.87	0.86 – 0.88	0.87	0.81 – 0.89	0.84
**75**	0.73 – 0.91	0.83	0.83 – 0.95	0.86	0.88 – 0.94	0.90	0.89 – 0.91	0.89
**125**	0.84 – 0.87	0.85	0.82 – 0.98	0.92	0.95 – 0.97	0.96	0.92 – 0.97	0.95
**175**	0.90 – 1.00	0.95	0.96 – 0.99	0.98	0.98 – 0.99	0.99	0.97 – 0.99	0.98
**225**	0.98 – 1.00	1.00	0.96 – 1.01	0.98	0.99 – 1.02	1.00	0.98 – 1.01	1.00
**All**	0.62 – 1.00	0.85	0.44 – 1.01	0.85	0.71 – 1.02	0.91	0.63 – 1.01	0.89

Table S3. Descriptive statistics (minimum, maximum, and mean) of GreenSeeker (GS) Normalized Difference Vegetative Index ($NDVI_{GS}$) vegetation index (VI) and Sufficiency-Index (SI) measured at the panicle initiation rice growth stage from N response trial sites established during the 2019 growing season.

$NDVI_{GS}$

Vegetation Index

Pre-plant N rate (kg N ha^{-1})	Arbuckle-19		Davis-19		Marysville-19		RES-19	
	**min – max**	**mean**	**min – max**	**mean**	**min – max**	**mean**	**min – max**	**mean**
**0**	0.18 – 0.28	0.23	0.38 – 0.51	0.47	0.45 – 0.52	0.48	0.31 – 0.43	0.37
**101**	0.58 – 0.60	0.59	0.38 – 0.55	0.48	0.68 – 0.69	0.68	0.60 – 0.66	0.63
**135**	0.61 – 0.66	0.64	0.56 – 0.59	0.57	0.70 – 0.71	0.70	0.60 – 0.70	0.66
**168**	0.62 – 0.72	0.68	0.51 – 0.67	0.58	0.72 – 0.75	0.73	0.53 – 0.69	0.63
**202**	0.65 – 0.73	0.70	0.56 – 0.70	0.62	0.72 – 0.75	0.74	0.58 – 0.72	0.68
**235**	0.68 – 0.75	0.72	0.59 – 0.64	0.61	0.73 – 0.76	0.75	0.61 – 0.75	0.67
**All**	0.18 – 0.75	0.59	0.38 – 0.70	0.55	0.45 – 0.76	0.68	0.31 – 0.75	0.61

Sufficiency-Index

Pre-plant N rate (kg N ha^{-1})	Arbuckle-19		Davis-19		Marysville-19		RES-19	
	**min – max**	**mean**	**min – max**	**mean**	**min – max**	**mean**	**min – max**	**mean**
**0**	0.25 – 0.38	0.32	0.58 – 0.77	0.70	0.59 – 0.69	0.63	0.43 – 0.59	0.52
**101**	0.79 – 0.83	0.80	0.57 – 0.84	0.72	0.90 – 0.92	0.91	0.84 – 0.92	0.88
**135**	0.83 – 0.91	0.87	0.84 – 0.89	0.86	0.92 – 0.95	0.94	0.84 – 0.98	0.93
**168**	0.84 – 0.98	0.93	0.77 – 1.01	0.88	0.95 – 1.00	0.97	0.74 – 0.97	0.88
**202**	0.89 – 0.99	0.95	0.85 – 1.05	0.93	0.95 – 1.00	0.99	0.82 – 1.00	0.95
**235**	0.93 – 1.02	0.98	0.89 – 0.96	0.92	0.97 – 1.00	0.99	0.85 – 1.05	0.93
**All**	0.25 – 1.02	0.81	0.57 – 1.05	0.83	0.59 – 1.00	0.90	0.43 – 1.05	0.85

Table S4. Descriptive statistics (minimum, maximum, and mean) of unmanned aerial system (UAS) Normalized Difference Red-Edge Index ($NDRE_{UAS}$) vegetation index (VI) and Sufficiency-Index (SI) measured at the panicle initiation rice growth stage from N response trial sites established during the 2019 growing season.

$NDRE_{UAS}$

Vegetation Index

**Pre-plant N rate (kg N ha^{-1})**	**Arbuckle-19**		**Davis-19**		**Marysville-19**		**RES-19**	
	**min – max**	**mean**	**min – max**	**mean**	**min – max**	**mean**	**min – max**	**mean**
**0**	0.33 – 0.35	0.35	0.31 – 0.39	0.34	0.35 – 0.42	0.39	0.43 – 0.46	0.45
**101**	0.49 – 0.53	0.52	0.40 – 0.42	0.41	0.55 – 0.59	0.57	0.53 – 0.58	0.56
**135**	0.54 – 0.58	0.57	0.37 – 0.48	0.44	0.59 – 0.61	0.60	0.49 – 0.62	0.57
**168**	0.57 – 0.61	0.59	0.45 – 0.53	0.48	0.58 – 0.64	0.62	0.55 – 0.63	0.59
**202**	0.59 – 0.62	0.61	0.41 – 0.54	0.48	0.62 – 0.66	0.64	0.54 – 0.64	0.60
**235**	0.60 – 0.65	0.63	0.47 – 0.52	0.50	0.64 – 0.67	0.66	0.53 – 0.66	0.59
**All**	0.33 – 0.65	0.54	0.31 – 0.54	0.44	0.35 – 0.67	0.58	0.43 – 0.66	0.56

Sufficiency-Index

**Pre-plant N rate (kg N ha^{-1})**	**Arbuckle-19**		**Davis-19**		**Marysville-19**		**RES-19**	
	**min – max**	**mean**	**min – max**	**mean**	**min – max**	**mean**	**min – max**	**mean**
**0**	0.52 – 0.55	0.54	0.59 – 0.73	0.64	0.53 – 0.64	0.59	0.67 – 0.73	0.70
**101**	0.77 – 0.83	0.81	0.74 – 0.80	0.78	0.84 – 0.89	0.86	0.83 – 0.91	0.88
**135**	0.85 – 0.91	0.88	0.70 – 0.90	0.82	0.89 – 0.93	0.91	0.77 – 0.98	0.89
**168**	0.90 – 0.96	0.93	0.85 – 1.00	0.91	0.88 – 0.98	0.94	0.86 – 0.99	0.92
**202**	0.92 – 0.97	0.95	0.78 – 1.01	0.91	0.94 – 0.99	0.98	0.84 – 1.00	0.93
**235**	0.94 – 1.01	0.98	0.89 – 0.98	0.94	0.96 – 1.01	0.99	0.83 – 1.03	0.93
**All**	0.52 – 1.01	0.85	0.59 – 1.01	0.83	0.53 – 1.01	0.88	0.67 – 1.03	0.88
